# Supplementary figures and images for: eIF2A represses cell wall biogenesis gene expression in Saccharomyces cerevisiae
Source: PLoS One. 2023 Nov 27;18(11):e0293228. doi: 10.1371/journal.pone.0293228 (PMC10681259; doi:10.1371/journal.pone.0293228)

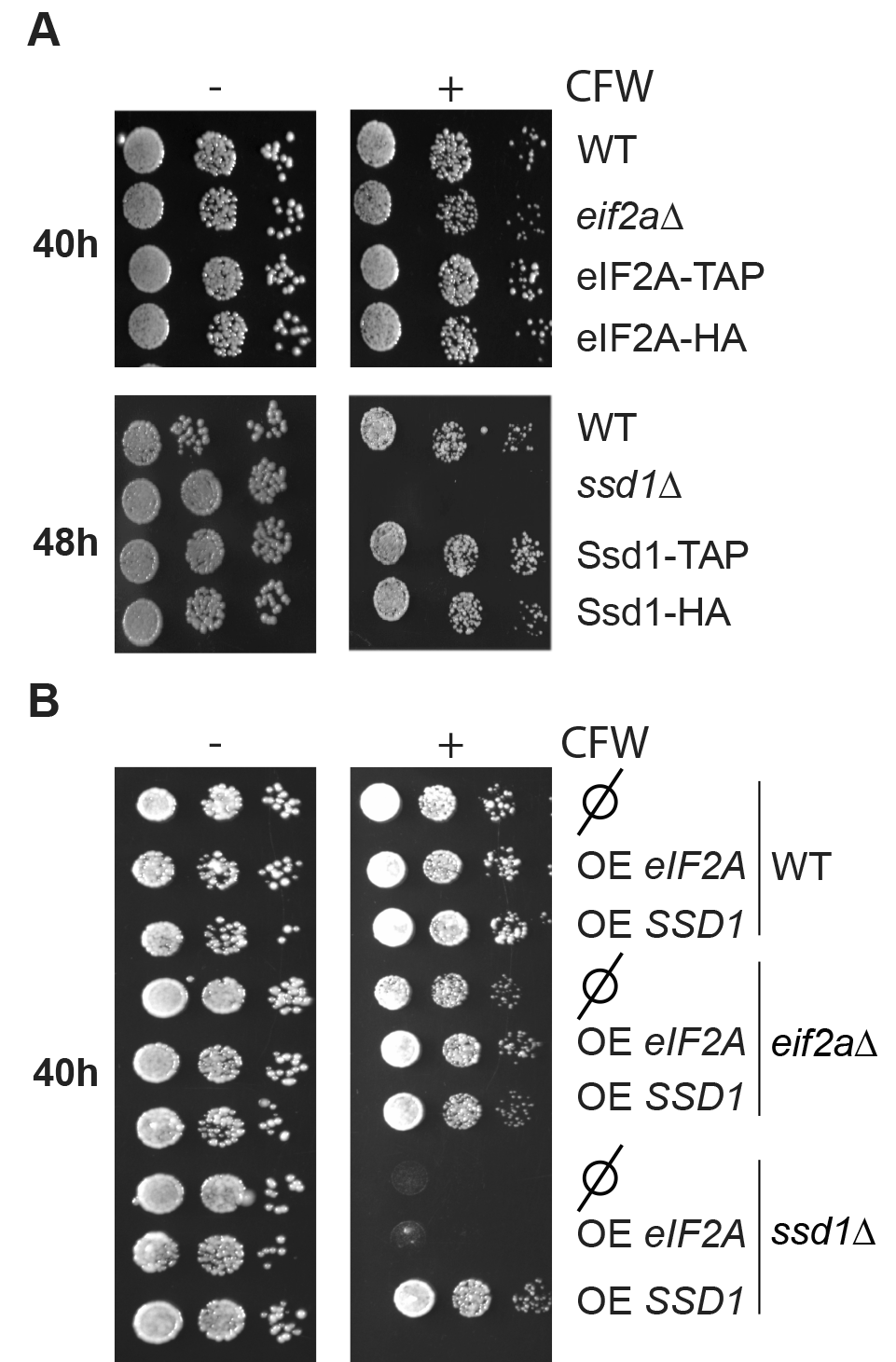

Supplement: S1 Fig — (A) Wild-type, deletion mutants and strains expressing tagged proteins were plated in 10−1 dilution series on rich medium with or without CFW and incubated at 30°C for 48 hours. (B) The CFW-sensitive phenotype of the eif2aΔ or ssd1Δ mutants were fully restored when eIF2A or Ssd1 was trans-expressed in the eif2aΔ or ssd1Δ mutants, respectively. Wild-type strain and the eif2aΔ or ssd1Δ mutants harboring either empty pCM190 (ø), pCM190: eIF2A (OE eIF2A) or pCM190: SSD1 (OE SSD1) vectors, were serially diluted and spotted on YPGlu rich medium supplemented with doxycycline and CFW (+) or not (-). (TIF) [file pone.0293228.s001.tif]

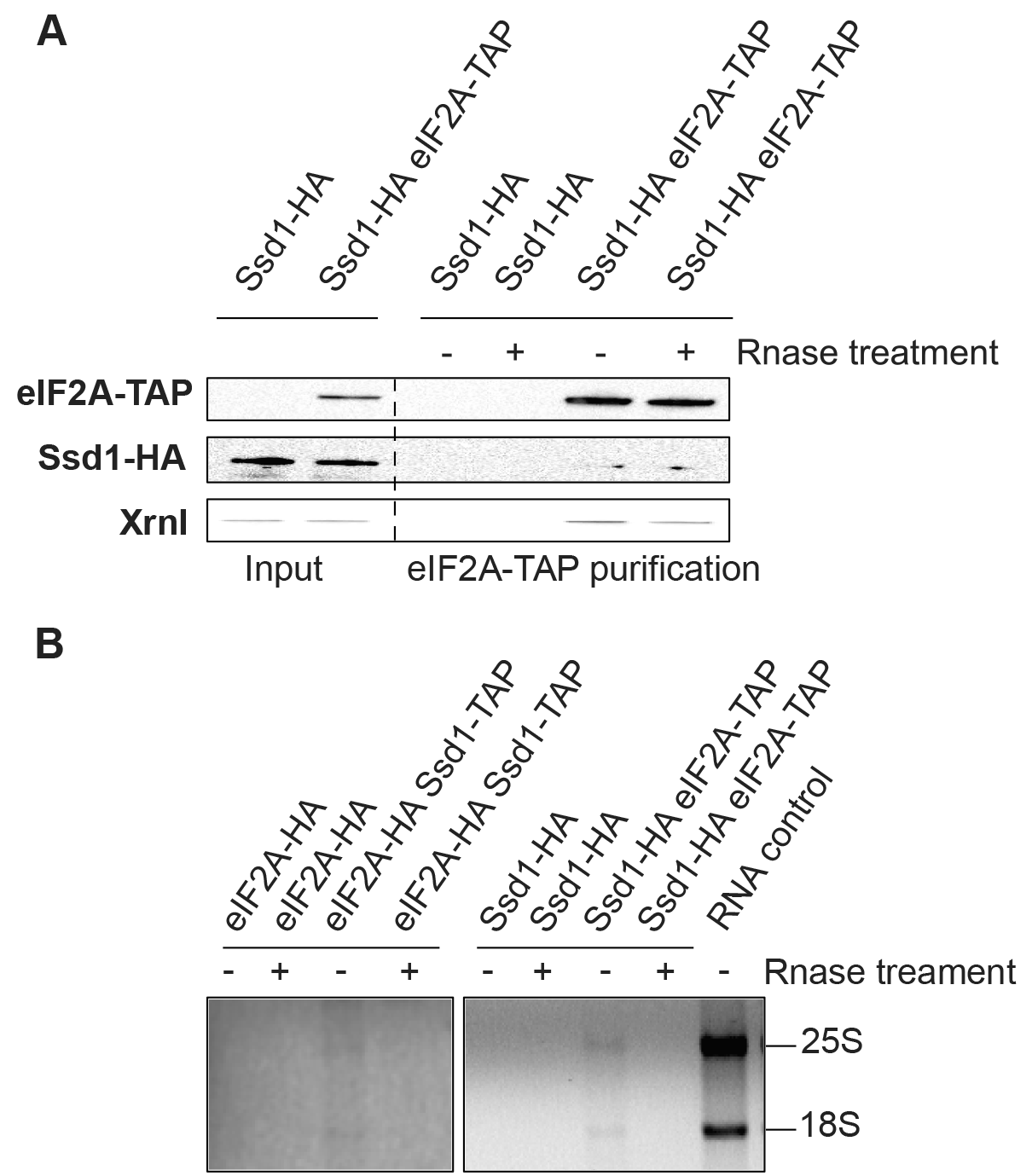

Supplement: S2 Fig — (A) Cells expressing eIF2A-TAP and Ssd1-HA proteins were cultivated until exponential-growth phase and eIF2A-TAP and its interaction partners were purified. eIF2A-associated complex was eluted after a nuclease treatment (+) or not (-) using micrococcal nuclease. A strain lacking the TAP-tag fused to the eIF2A protein was used as a control. Total (input) as well as purified proteins were separated on a polyacrylamide gel and TAP-, HA-tagged and XrnI proteins were revealed by Western Blot with PAP, anti-HA or anti-XrnI antibodies, respectively. (B) RNA was extracted from Ssd1- or eIF2A-associated complexes and separated by agarose gel electrophoresis. As a control, 1 μg of RNA from the wild-type strain was loaded to visualize 25S and 18S ribosomal RNA. (TIF) [file pone.0293228.s002.tif]

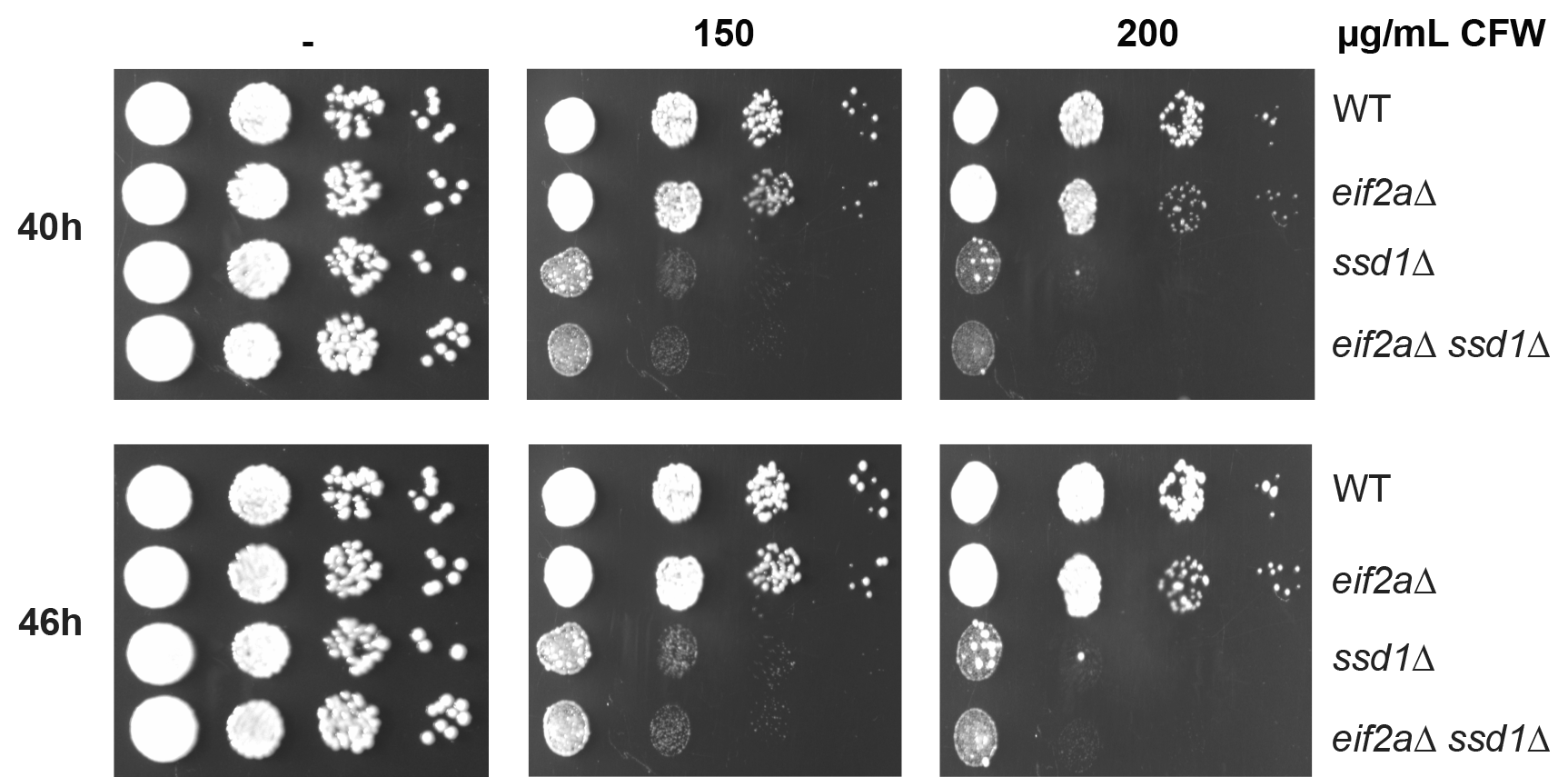

Supplement: S3 Fig — Wild-type, ssd1Δ, eif2aΔ strains and the ssd1Δ eif2aΔ double mutant were serially diluted and spotted on YPGlu rich medium supplemented or not with CFW at the indicated concentrations. (TIF) [file pone.0293228.s003.tif]

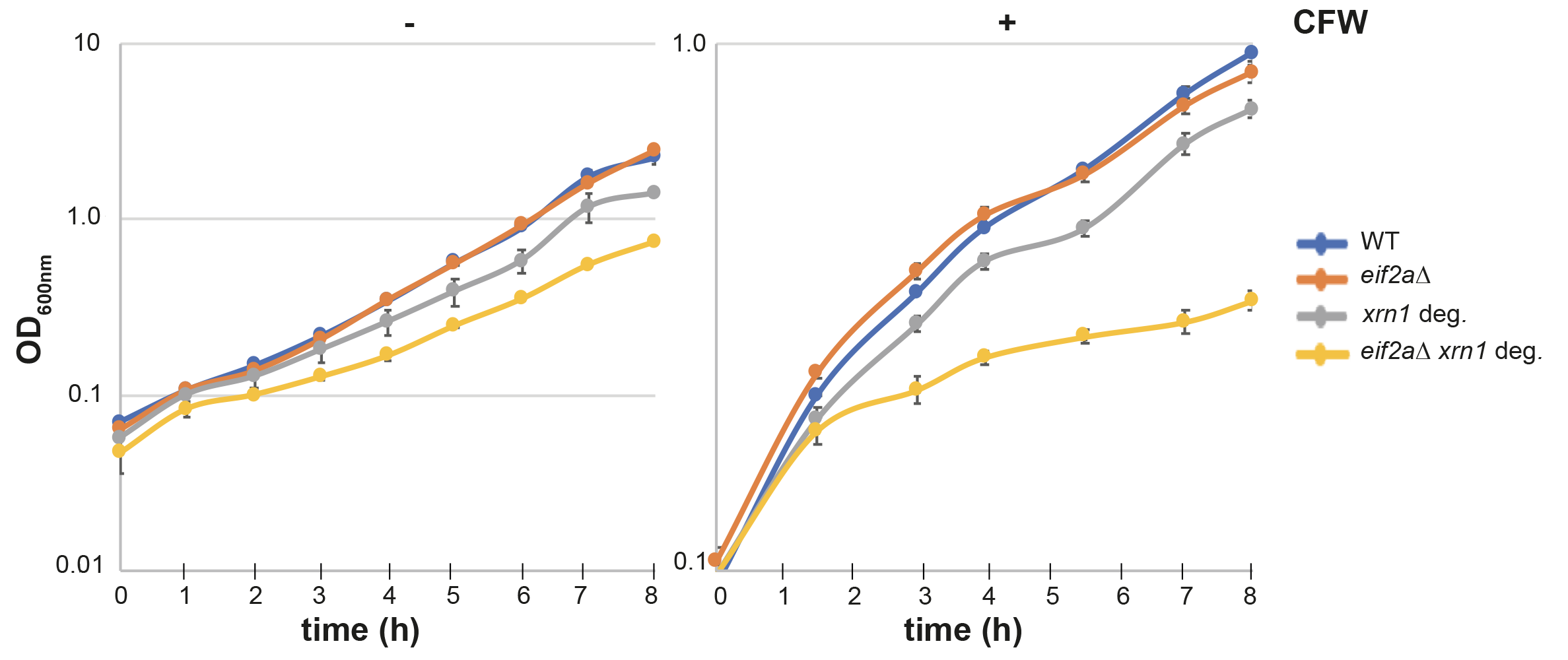

Supplement: S4 Fig — Wild-type, xrn1-deg, eif2aΔ mutant and xrn1-deg eif2aΔ double mutant were cultivated in YPGlu medium until exponential-growth phase. IAA was added at a final concentration of 100 μM and CFW was added or not at a final concentration of 500 μg/ml. OD600nm was taken at indicated times. Error bars indicate the standard deviations of averages for at least three independent experiments. (TIF) [file pone.0293228.s004.tif]
