# Supplementary material for: eIF2A represses cell wall biogenesis gene expression in Saccharomyces cerevisiae
Source: PLoS One. 2023 Nov 27;18(11):e0293228. doi: 10.1371/journal.pone.0293228 (PMC10681259; doi:10.1371/journal.pone.0293228)
Supplement: S1 Table — (DOCX) [file pone.0293228.s005.docx]

**Supporting information**

**S1 Table. List of the *S. cerevisiae* strains used in this study**

| **Strains** | **Genotypes** | **References** |
| --- | --- | --- |
| BY4741 | *MATa, ura3∆0, his3∆1, leu2∆0, met15∆0* | [1] |
| LMA4742 | as *BY4741*, ***Xrn1*-*deg***:*KANMX6* | This study |
| LMA5539  LMA1393  LMA5431 | as *BY4741*, ***Xrn1*-*deg***:*KANMX6,* ***eif2a****∆::HYGMX6*  as *BY4741*, ***xrn1****∆:KANMX4*  as *BY4741*, ***eIF2a-TAP****:KANMX6* | This study  Euroscarf  This study |
| LMA5435 | as *BY4741*, ***eIF2a-TAP****:HYGMX6* | This study |
| LMA5535 | as *BY4741,* ***eIF2a-TAP****:HYGMX6, xrn1D206A* | This study |
| LMA5664 | as *BY4741*, ***Tos1-TAP****:HIS3MX6* | [2] |
| LMA5665 | as *BY4741*, ***Ccw14-TAP****:HIS3MX6* | [2] |
| LMA5749 | as *BY4741*, ***Sun4-TAP****:HIS3MX6* | [2] |
| LMA5755  LMA5839 | as *BY4741*, ***Sun4*-*TAP***:*HIS3*MX6*,* ***ssd1****∆::KANMX4*  as *BY4741*, ***Cln1-TAP****:HIS3MX6* | This study  [2] |
| LMA5684 | as *BY4741*, ***Ssd1-TAP****:HIS3MX6* | [2] |
| LMA5780 | as *BY4741*, ***eIF2a-3HA****:KANMX6* | This study |
| LMA5783 | as *BY4741*, ***eIF2A-3HA****:KANMX6,* ***Ssd1-TAP****:HIS3MX6* | This study |
| LMA5784 | as *BY4741*, ***Ssd1-3HA****:HIS3MX6* | This study |
| LMA5786 | as *BY4741*, ***eIF2a-TAP****:KANMX6,* ***Ssd1-3HA****:HIS3MX6* | This study |
| LMA5646  LMA5647  LMA5436 | as *BY4741*, ***hsp150****∆::KANMX4*  as *BY4741*, ***uth1****∆::KANMX4*  as *BY4741*, ***eif2a****∆::KANMX4* | Euroscarf  Euroscarf  Euroscarf |
| LMA5770 | as *BY4741*, ***eif2a****∆::HYGMX6* | This study |
| LMA5752 | as *BY4741*, ***ssd1****∆::KANMX4* | Euroscarf |
| LMA5774 | as *BY4741*, ***ssd1****∆:: HYGMX6* | This study |
| LMA5788 | as *BY4741*, ***eIF2a-TAP****:KANMX6,* ***ssd1****∆:: HYGMX6* | This study |
| LMA5778 | as *BY4741*, ***eif2a****∆::HYGMX6,* ***ssd1****∆::KANMX4* | This study |
|  |  |  |
