# Supplementary material for: eIF2A represses cell wall biogenesis gene expression in Saccharomyces cerevisiae
Source: PLoS One. 2023 Nov 27;18(11):e0293228. doi: 10.1371/journal.pone.0293228 (PMC10681259; doi:10.1371/journal.pone.0293228)
Supplement: S2 Table — (DOCX) [file pone.0293228.s006.docx]

**Supporting information**

|  |  |  |
| --- | --- | --- |

**S2 Table. List of the plasmids used in this study**

| **Plasmids** | **Markers** | **References** | |
| --- | --- | --- | --- |
| pCM190 | AMP, URA | ATCC |  |
| pCM190-*eIF2A* | AMP, URA | This study |  |
| pCM190-*SSD1* | AMP, URA | This study |  |
| pAG32 | AMP, Hyg | Addgene |  |
