# Supplementary material for: eIF2A represses cell wall biogenesis gene expression in Saccharomyces cerevisiae
Source: PLoS One. 2023 Nov 27;18(11):e0293228. doi: 10.1371/journal.pone.0293228 (PMC10681259; doi:10.1371/journal.pone.0293228)
Supplement: S3 Table — (DOCX) [file pone.0293228.s007.docx]

**Supporting information**

**S3 Table. List of the oligonucleotides used in this study**

| **Names** | **Sequences** | **Used for** |
| --- | --- | --- |
| MFR1172 | CGCGGATCCAGGTTCAATAACACCTAAAC | pCM190-e*IF2A* |
| MFR1173 | ATAGTTTAGCGGCCGCGATACATCAGTTTCTTC | pCM190-e*IF2A* |
| MFR1231 | CGCGGATCCCAATTATTCCATCTTTATAC | pCM190-*SSD1* |
| MFR1232 | ATAGTTTAGCGGCCGCCAATGACGATATTGGTAGAAG | pCM190-*SSD1* |
| AJ529 | GCTAATACGACTCACTATAGGG | T7 dble strand |
| MFR1233 | CCAAAGTTGCTTCTTCTTCTG | *SUN4* RNA Probe |
| MFR1234 | GCTAATACGACTCACTATAGGGGCCACCCGTAGAAGTGTCAG | *SUN4* RNA Probe |
| MFR1239 | CAACGAGCTAAATGGTGAAC | *CTS1* RNA Probe |
| MFR1240 | GCTAATACGACTCACTATAGGGGTGTTGTGGTGGTACCTAGAC | *CTS1* RNA Probe |
| MFR1263 | ACCTCGACTATCACGTCCAC | *SRL1* RNA Probe |
| MFR1264 | GCTAATACGACTCACTATAGGGTGCACCGGTGACAGTAAC | *SRL1* RNA Probe |
| GB1417 | GTTATCGTCAAAGCTAGATTCGTCTCCAAGTTGGCTGAAGAAAAAATCAGAG  CCCTATAGTGAGTCGTATTAGC | *RPL28* RNA Probe |
| MFR1144 | CTGAAGAAAAAGTGTTGAAAGATTTGGAAAAGTTGGGTTGGAAGGATGAA  CGGATCCCCGGGTTAATTAA | eIF2a-TAP, -HA |
| MFR1145 | GACGTTGTTAATATTTACACAGTTGTATGGATACATCAGTTTCTTCTAGT  GAATTCGAGCTCGTTTAAAC | eIF2a-TAP, -HA |
| MFR1225 | CTTTGCCATGTTTAACCGTCCGTGCATTAAATCCATTCATGAAGAGGGTA  CGGATCCCCGGGTTAATTAA | Ssd1-HA |
| MFR1226 | ACGAAAGTGAAAAACAAGAAAAACAGCAATGACGATATTGGTAGAAGAGA  GAATTCGAGCTCGTTTAAAC | Ssd1-HA |
| MFR1215 | TTCAGCGCAAAGATTTGGC | *ssd1∆* ::Kan |
| MFR1216 | CCGGAACGTGGATTAAAAGG | *ssd1∆* ::Kan |
| MFR1147 | AATCCGCCAAGAAGAGACG | *eif2a∆* ::Hyg |
| MFR1148 | CAGACGACTAGACATAGCGAG | *eif2a∆* ::Hyg |
