# Supplementary material for: eIF2A represses cell wall biogenesis gene expression in Saccharomyces cerevisiae
Source: PLoS One. 2023 Nov 27;18(11):e0293228. doi: 10.1371/journal.pone.0293228 (PMC10681259; doi:10.1371/journal.pone.0293228)
Supplement: S4 Table — (DOCX) [file pone.0293228.s008.docx]

**Supporting information**

**S4 Table. List of the antibodies used in this study**

| **Targets** | **Antibodies** | **Dilutions** |
| --- | --- | --- |
| TAP-tagged | PAP (Peroxydase anti-Peroxidase complex), Sigma | 1/5,000 |
| HA-tagged | Anti-HA perixidase High affinity (3F10) Roche | 1/2,000 |
| G6PDH | Rabbit Polyclonal | 1/100,000 |
| Xrn1 | Gift from A. Jonhson | 1/2,000 |
| DIG RNA | Anti-digoxigenin-POD, Fab fragments, Roche | 1/2,000 |
