# Supplementary material for: eIF2A represses cell wall biogenesis gene expression in Saccharomyces cerevisiae
Source: PLoS One. 2023 Nov 27;18(11):e0293228. doi: 10.1371/journal.pone.0293228 (PMC10681259; doi:10.1371/journal.pone.0293228)
Supplement: S1 File — (DOCX) [file pone.0293228.s013.docx]

**Supplemental informations**

**S1 Table. List of the *S. cerevisiae* strains used in this study**

| **Strains** | **Genotypes** | **References** |
| --- | --- | --- |
| BY4741 | *MATa, ura3∆0, his3∆1, leu2∆0, met15∆0* | [1] |
| LMA4742 | as *BY4741*, ***Xrn1*-*deg***:*KANMX6* | This study |
| LMA5539  LMA1393  LMA5431 | as *BY4741*, ***Xrn1*-*deg***:*KANMX6,* ***eif2a****∆::HYGMX6*  as *BY4741*, ***xrn1****∆:KANMX4*  as *BY4741*, ***eIF2a-TAP****:KANMX6* | This study  Euroscarf  This study |
| LMA5435 | as *BY4741*, ***eIF2a-TAP****:HYGMX6* | This study |
| LMA5535 | as *BY4741,* ***eIF2a-TAP****:HYGMX6, xrn1D206A* | This study |
| LMA5664 | as *BY4741*, ***Tos1-TAP****:HIS3MX6* | [2] |
| LMA5665 | as *BY4741*, ***Ccw14-TAP****:HIS3MX6* | [2] |
| LMA5749 | as *BY4741*, ***Sun4-TAP****:HIS3MX6* | [2] |
| LMA5755  LMA5839 | as *BY4741*, ***Sun4*-*TAP***:*HIS3*MX6*,* ***ssd1****∆::KANMX4*  as *BY4741*, ***Cln1-TAP****:HIS3MX6* | This study  [2] |
| LMA5684 | as *BY4741*, ***Ssd1-TAP****:HIS3MX6* | [2] |
| LMA5780 | as *BY4741*, ***eIF2a-3HA****:KANMX6* | This study |
| LMA5783 | as *BY4741*, ***eIF2A-3HA****:KANMX6,* ***Ssd1-TAP****:HIS3MX6* | This study |
| LMA5784 | as *BY4741*, ***Ssd1-3HA****:HIS3MX6* | This study |
| LMA5786 | as *BY4741*, ***eIF2a-TAP****:KANMX6,* ***Ssd1-3HA****:HIS3MX6* | This study |
| LMA5646  LMA5647  LMA5436 | as *BY4741*, ***hsp150****∆::KANMX4*  as *BY4741*, ***uth1****∆::KANMX4*  as *BY4741*, ***eif2a****∆::KANMX4* | Euroscarf  Euroscarf  Euroscarf |
| LMA5770 | as *BY4741*, ***eif2a****∆::HYGMX6* | This study |
| LMA5752 | as *BY4741*, ***ssd1****∆::KANMX4* | Euroscarf |
| LMA5774 | as *BY4741*, ***ssd1****∆:: HYGMX6* | This study |
| LMA5788 | as *BY4741*, ***eIF2a-TAP****:KANMX6,* ***ssd1****∆:: HYGMX6* | This study |
| LMA5778 | as *BY4741*, ***eif2a****∆::HYGMX6,* ***ssd1****∆::KANMX4* | This study |
|  |  |  |

**S2 Table. List of the plasmids used in this study**

| **Plasmids** | **Markers** | **References** | |
| --- | --- | --- | --- |
| pCM190 | AMP, URA | ATCC |  |
| pCM190-*eIF2A* | AMP, URA | This study |  |
| pCM190-*SSD1* | AMP, URA | This study |  |
| pAG32 | AMP, Hyg | Addgene |  |

**S3 Table. List of the oligonucleotides used in this study**

| **Names** | **Sequences** | **Used for** |
| --- | --- | --- |
| MFR1172 | CGCGGATCCAGGTTCAATAACACCTAAAC | pCM190-e*IF2A* |
| MFR1173 | ATAGTTTAGCGGCCGCGATACATCAGTTTCTTC | pCM190-e*IF2A* |
| MFR1231 | CGCGGATCCCAATTATTCCATCTTTATAC | pCM190-*SSD1* |
| MFR1232 | ATAGTTTAGCGGCCGCCAATGACGATATTGGTAGAAG | pCM190-*SSD1* |
| AJ529 | GCTAATACGACTCACTATAGGG | T7 dble strand |
| MFR1233 | CCAAAGTTGCTTCTTCTTCTG | *SUN4* RNA Probe |
| MFR1234 | GCTAATACGACTCACTATAGGGGCCACCCGTAGAAGTGTCAG | *SUN4* RNA Probe |
| MFR1239 | CAACGAGCTAAATGGTGAAC | *CTS1* RNA Probe |
| MFR1240 | GCTAATACGACTCACTATAGGGGTGTTGTGGTGGTACCTAGAC | *CTS1* RNA Probe |
| MFR1263 | ACCTCGACTATCACGTCCAC | *SRL1* RNA Probe |
| MFR1264 | GCTAATACGACTCACTATAGGGTGCACCGGTGACAGTAAC | *SRL1* RNA Probe |
| GB1417 | GTTATCGTCAAAGCTAGATTCGTCTCCAAGTTGGCTGAAGAAAAAATCAGAG  CCCTATAGTGAGTCGTATTAGC | *RPL28* RNA Probe |
| MFR1144 | CTGAAGAAAAAGTGTTGAAAGATTTGGAAAAGTTGGGTTGGAAGGATGAA  CGGATCCCCGGGTTAATTAA | eIF2a-TAP, -HA |
| MFR1145 | GACGTTGTTAATATTTACACAGTTGTATGGATACATCAGTTTCTTCTAGT  GAATTCGAGCTCGTTTAAAC | eIF2a-TAP, -HA |
| MFR1225 | CTTTGCCATGTTTAACCGTCCGTGCATTAAATCCATTCATGAAGAGGGTA  CGGATCCCCGGGTTAATTAA | Ssd1-HA |
| MFR1226 | ACGAAAGTGAAAAACAAGAAAAACAGCAATGACGATATTGGTAGAAGAGA  GAATTCGAGCTCGTTTAAAC | Ssd1-HA |
| MFR1215 | TTCAGCGCAAAGATTTGGC | *ssd1∆* ::Kan |
| MFR1216 | CCGGAACGTGGATTAAAAGG | *ssd1∆* ::Kan |
| MFR1147 | AATCCGCCAAGAAGAGACG | *eif2a∆* ::Hyg |
| MFR1148 | CAGACGACTAGACATAGCGAG | *eif2a∆* ::Hyg |

**S4 Table. List of the antibodies used in this study**

| **Targets** | **Antibodies** | **Dilutions** |
| --- | --- | --- |
| TAP-tagged | PAP (Peroxidase anti-Peroxidase complex), Sigma | 1/5,000 |
| HA-tagged | Anti-HA Peroxidase High affinity (3F10) Roche | 1/2,000 |
| G6PDH | Rabbit Polyclonal | 1/100,000 |
| Xrn1 | Gift from A. Jonhson | 1/2,000 |
| DIG RNA | Anti-digoxigenin-POD, Fab fragments, Roche | 1/2,000 |

**S5 Table. List of the eIF2A mRNAs targets**

The functions are provided by SGD <https://www.yeastgenome.org/>

**References**

1. Brachmann CB, Davies A, Cost GJ, Caputo E, Li J, Hieter P, et al. Designer deletion strains derived from Saccharomyces cerevisiae S288C: a useful set of strains and plasmids for PCR-mediated gene disruption and other applications. Yeast. 1998;14: 115–132. doi:10.1002/(SICI)1097-0061(19980130)14:2<115::AID-YEA204>3.0.CO;2-2

2. Ghaemmaghami S, Huh W-K, Bower K, Howson RW, Belle A, Dephoure N, et al. Global analysis of protein expression in yeast. Nature. 2003;425: 737–741. doi:10.1038/nature02046
