# Supplementary material for: eIF2A represses cell wall biogenesis gene expression in Saccharomyces cerevisiae
Source: PLoS One. 2023 Nov 27;18(11):e0293228. doi: 10.1371/journal.pone.0293228 (PMC10681259; doi:10.1371/journal.pone.0293228)
Supplement: S1 Raw images — (PDF) [file pone.0293228.s014.pdf]

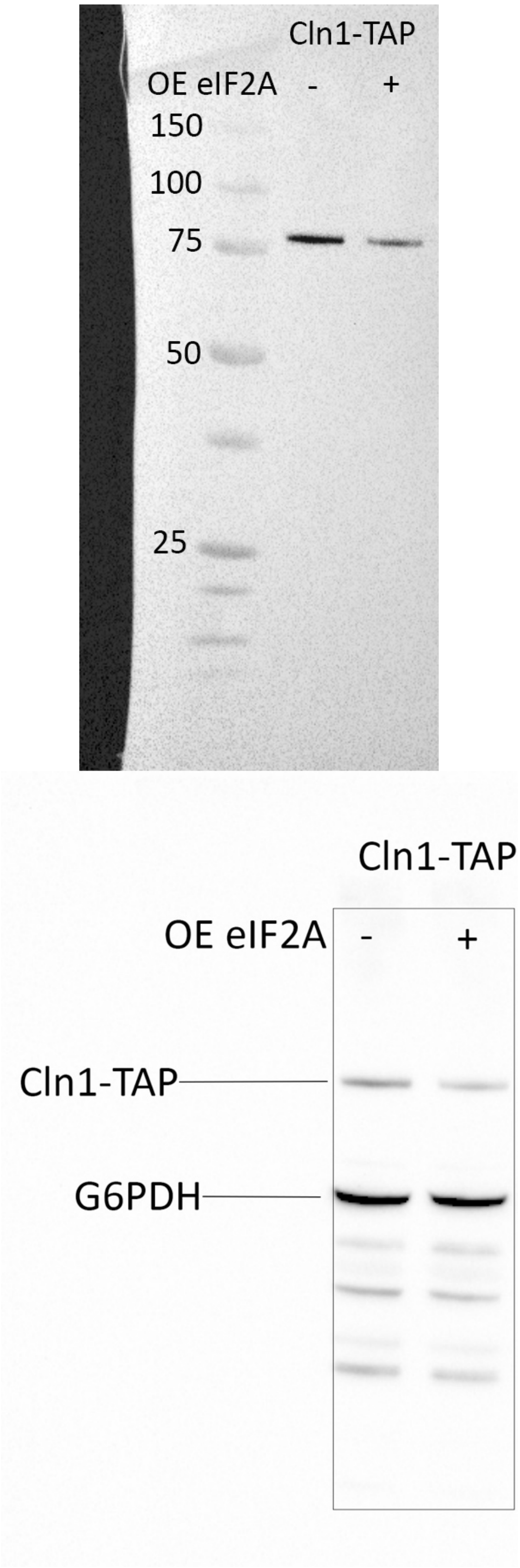
Cln1-TAP
OE eIF2A - +
150
100
75
50
25
Cln1-TAP
OE eIF2A - +
Cln1-TAP
G6PDH

Figure 3

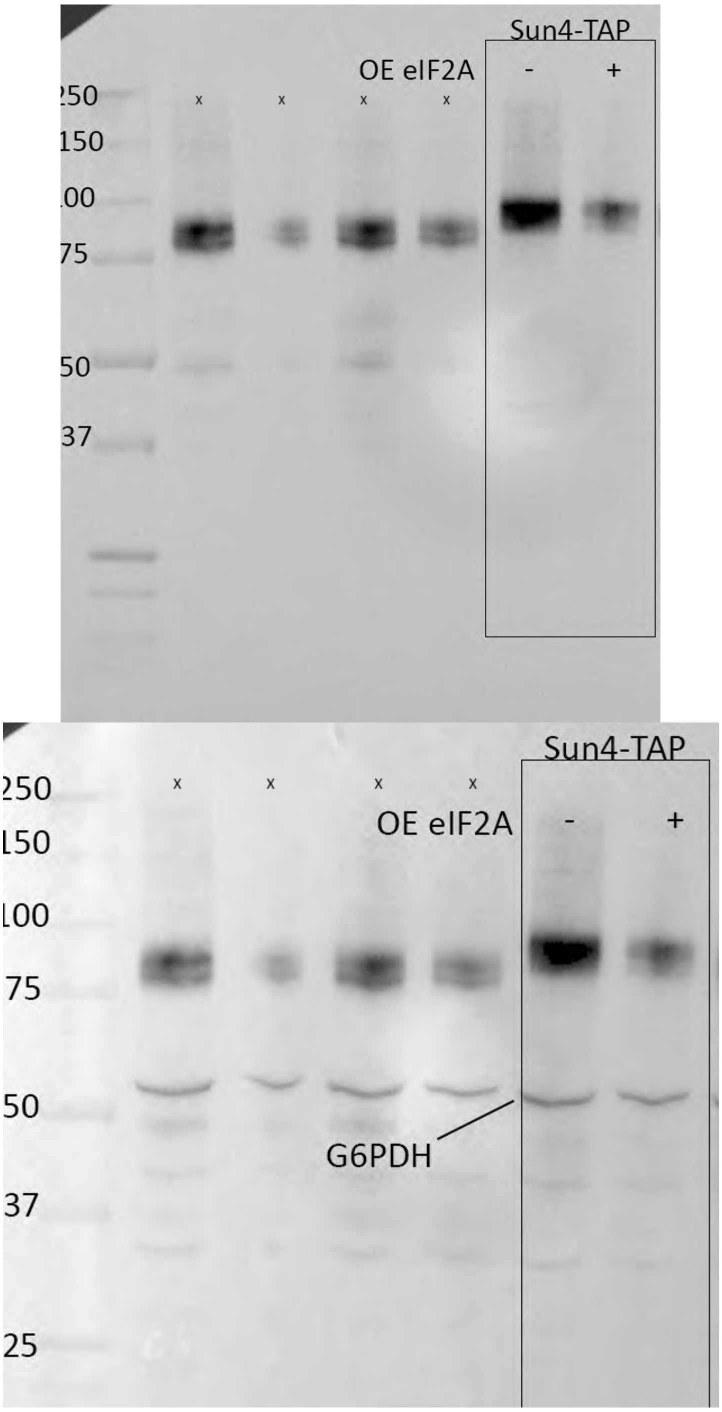
Sun4-TAP
OE eIF2A
-
+
250
150
100
75
50
37
x
x
x
x
Sun4-TAP
OE eIF2A
-
+
250
150
100
75
50
37
25
G6PDH

Figure 3

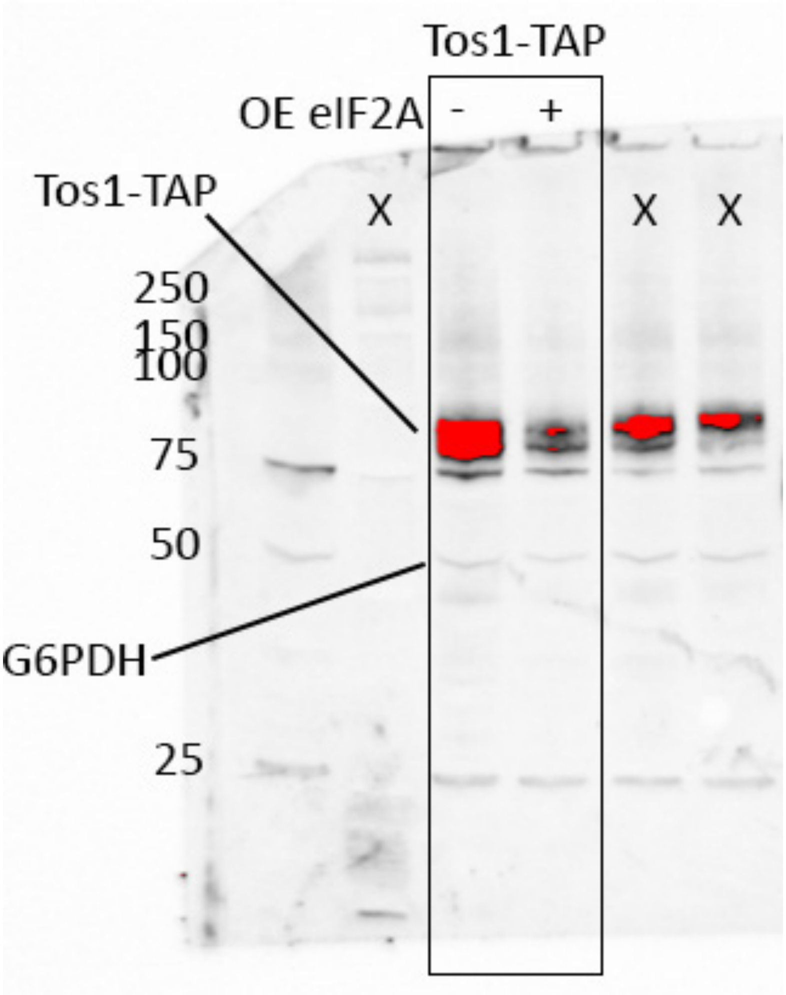

Figure 3

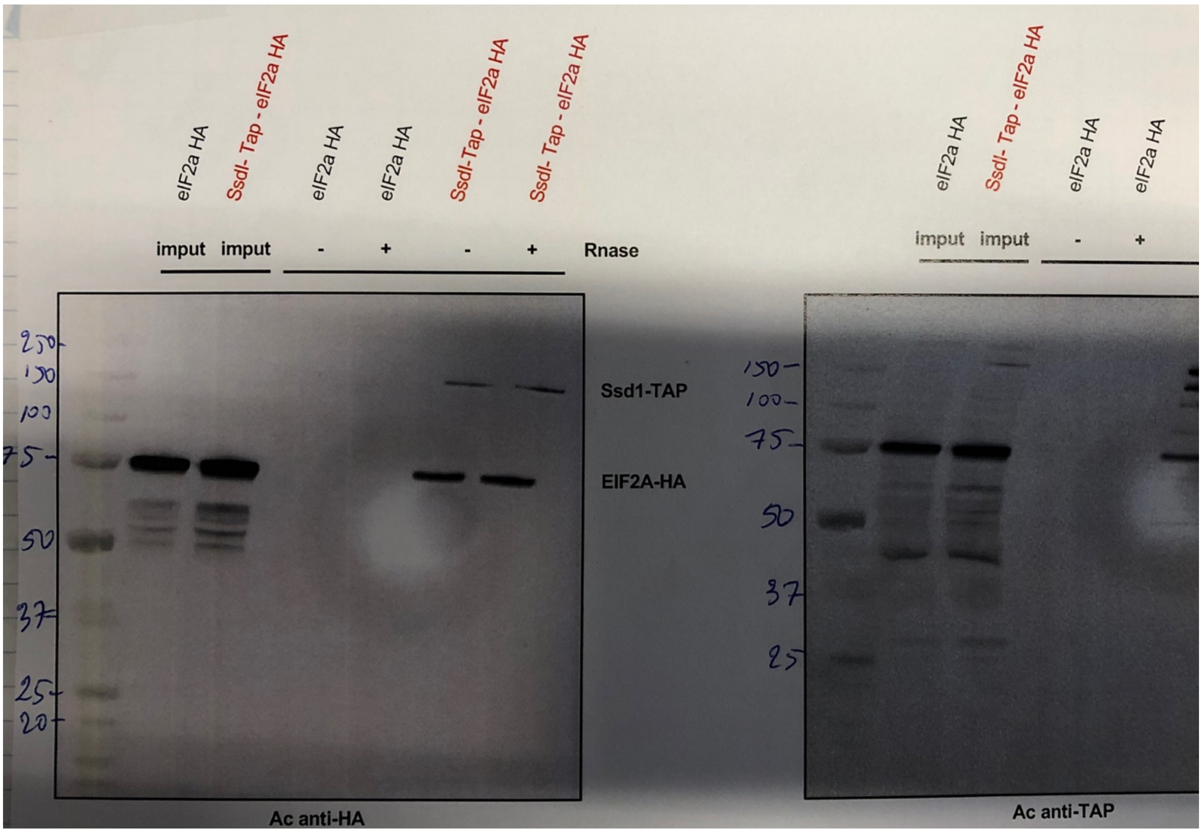

Figure 5C

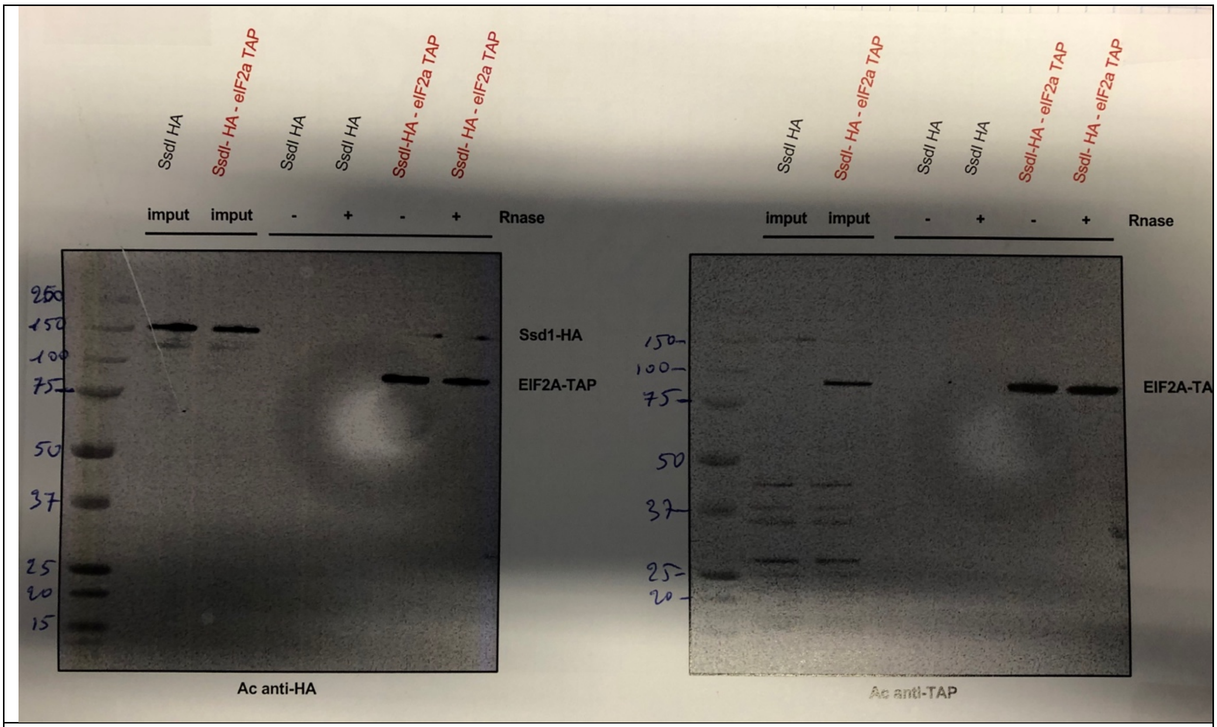

Figure S2

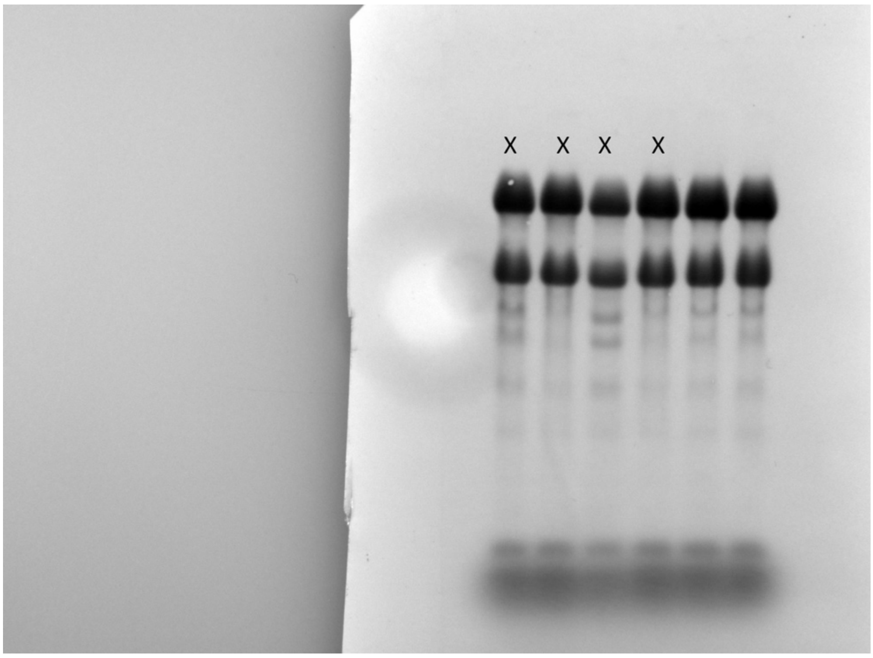

Figure 6 methylene blue input 18S and 25S

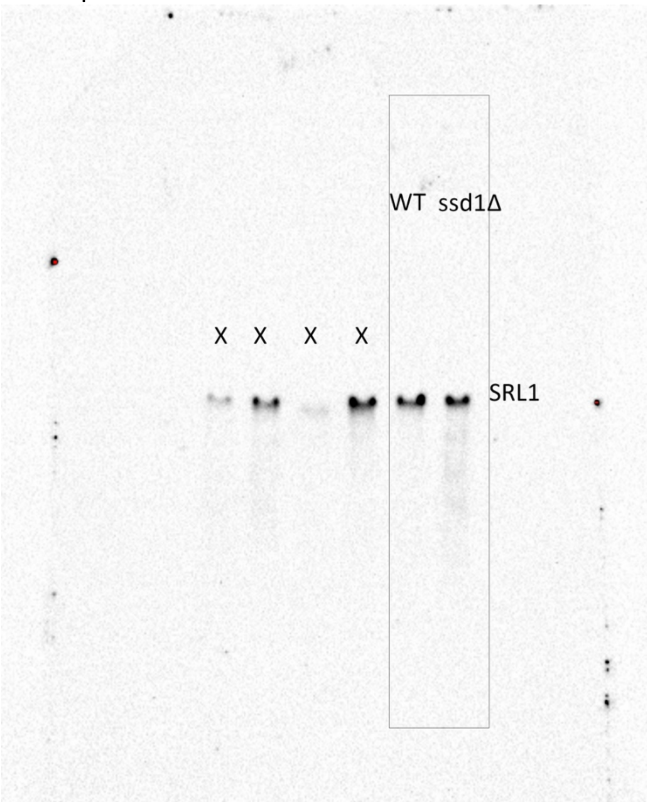

Figure 6 input SRL1 RNA Probe

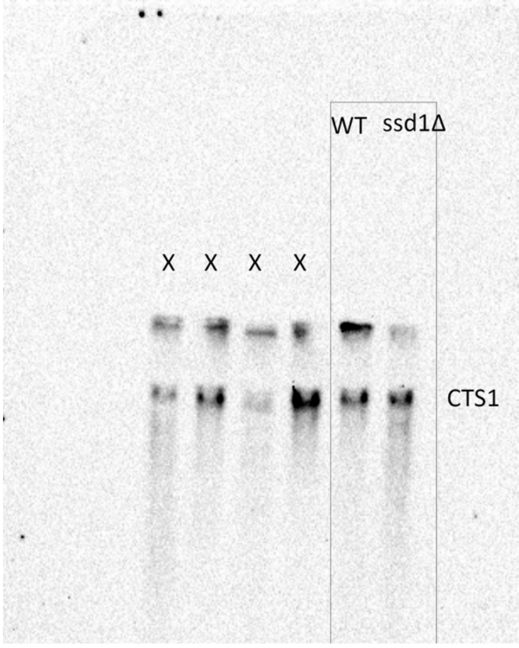

Figure 6 input CTS1 RNA probe

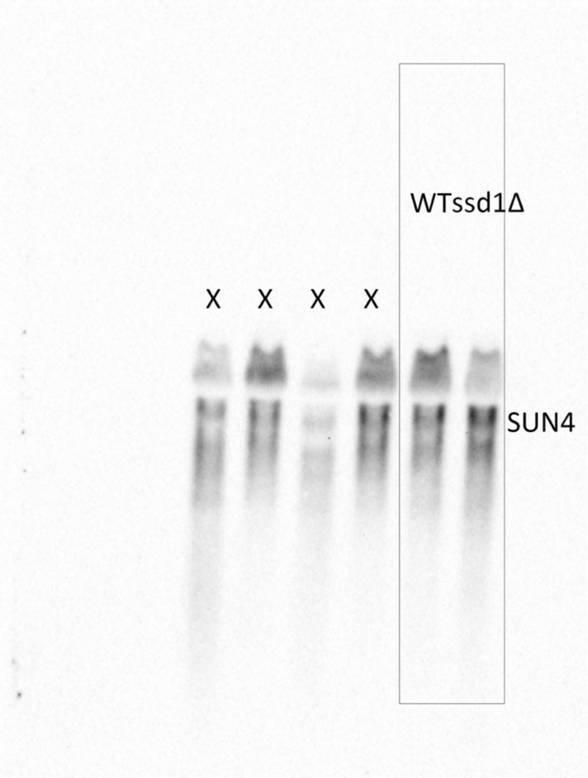

Figure 6 input SUN4 RNA probe

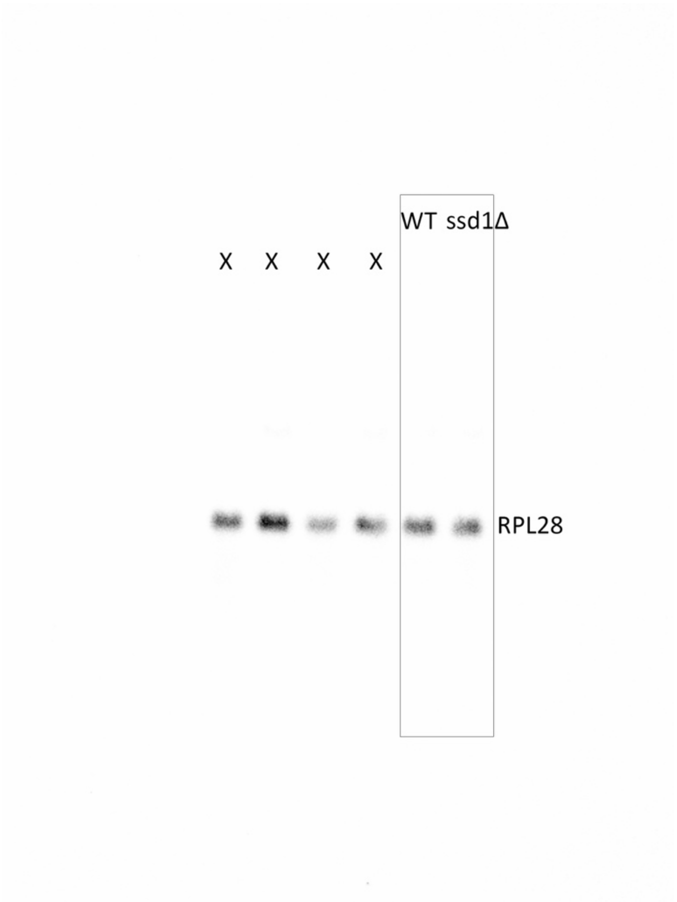

Figure 6 input RPL28 RNA probe

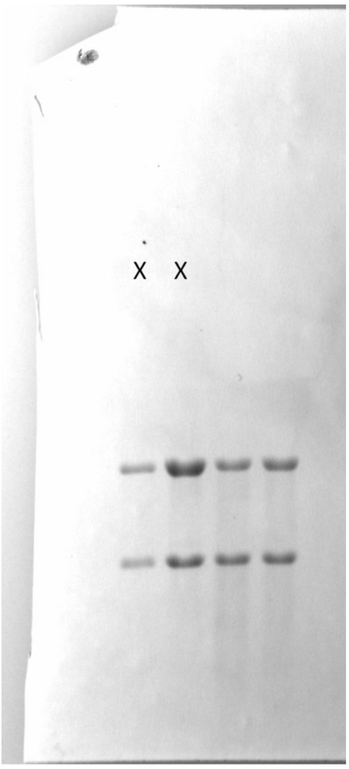

Figure 6 methylene blue RIP

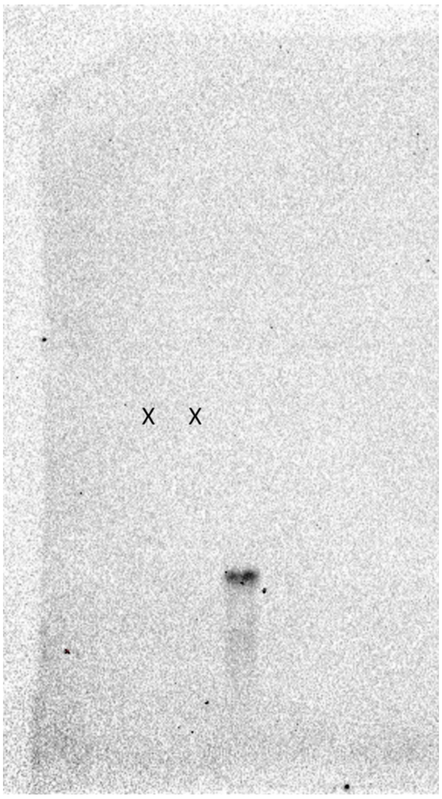

Figure 6 RIP SUN4 RNA probe

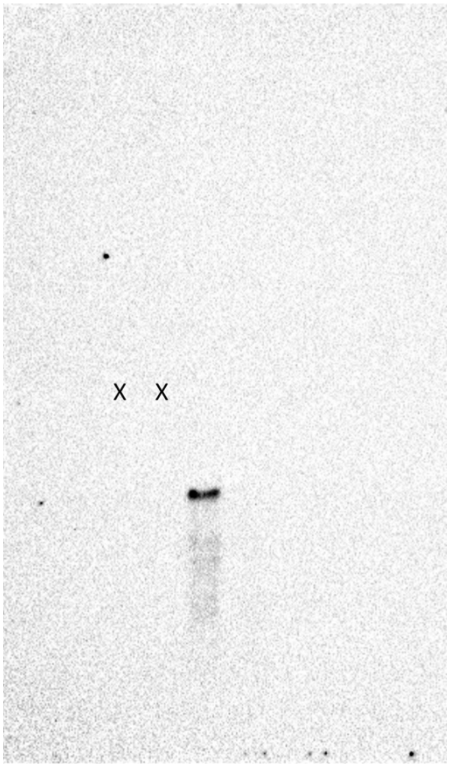

Figure 6 RIP CTS1 RNA probe

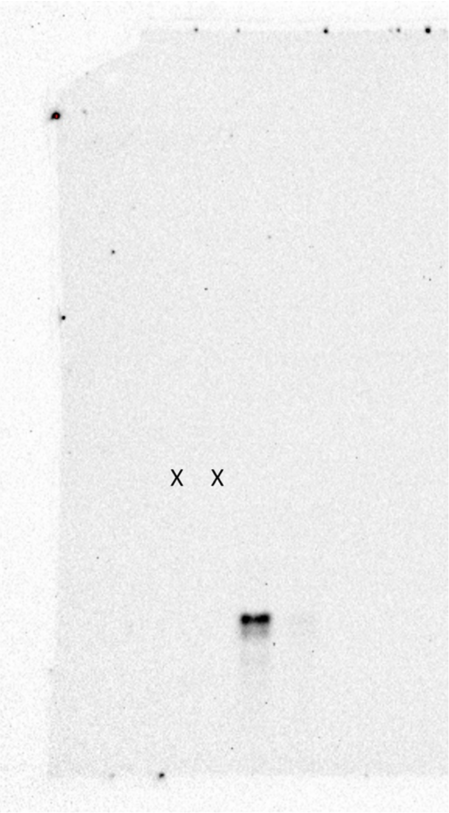

Figure 6 RIP SRL1 RNA probe

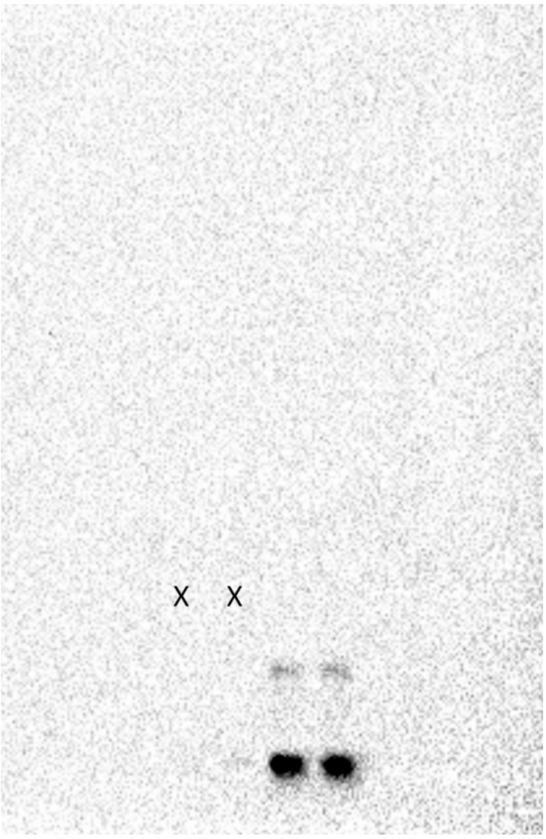

Figure 6 RIP RPL28 RNA probe

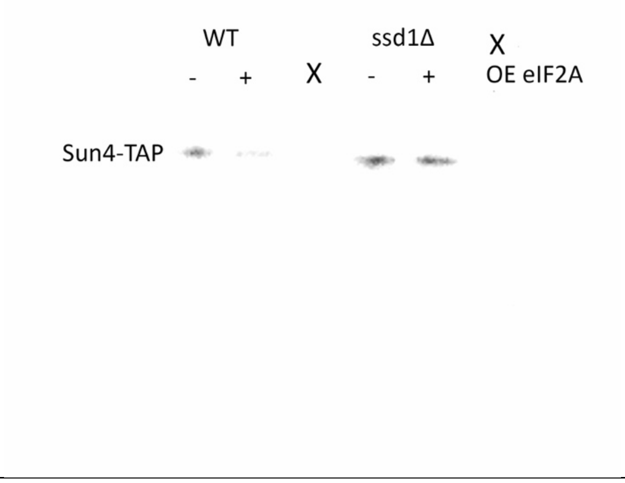

Figure 7 Western PAP hybridization

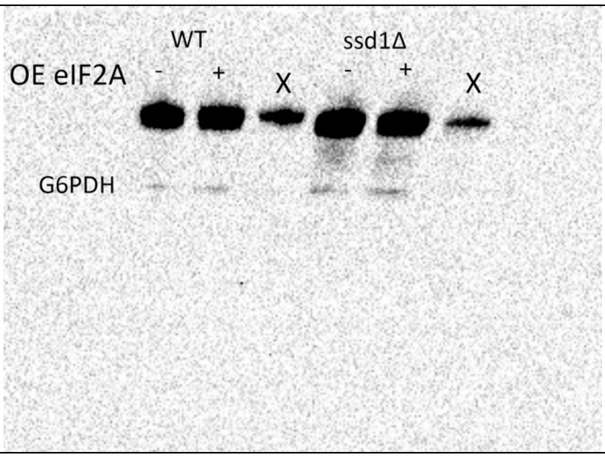

Figure 7 western anti G6PDH hybridization
