# Supplementary material for: eIF2A represses cell wall biogenesis gene expression in Saccharomyces cerevisiae
Source: PLoS One. 2023 Nov 27;18(11):e0293228. doi: 10.1371/journal.pone.0293228 (PMC10681259; doi:10.1371/journal.pone.0293228)
Supplement: Attachment — Submitted filename: Response to Reviewers.docx [file pone.0293228.s016.docx]

Dear editor,

We are pleased to see that the referees and you think that our manuscript deserves a publication in PLOS ONE. Please find enclosed a second version, revised according to the questions raised by the reviewer 2.

Point 1:

The statistical significance values calculated for Figures 3 and 7 do not need multiple hypothesis testing correction because they were applied to a small number of samples.

For the transcriptomic data, the classical multiple hypothesis testing correction method was done by DESeq2.

For the proteomic analysis, the multiple hypothesis testing correction method was done by the Perseus software. We added “implemented in Perseus” line 302 and together with the reference 36.

Point 2:

We agree with the Review 2 that there is a contradiction between the text and the rebuttal letter on this point.

We deleted this part of the sentence line 293 “in the presence of a vast number of background binding proteins”

As suggested by the reviewer 2, we added information about the number of proteins identified and quantified: line 433-435

A total number of 1509 proteins were identified in either input or purified samples but only 1037 were quantified in both. The enrichment level for each protein is indicated in the S3 Dataset.

Additional point:

We detected an error in the Material and methods related to the Western blot method. We replaced “Positively Charged Nylon Membrane (BrightStar^TM^.Plus Invitrogen)” by “Nitrocellulose Membranes 0.45µm (Bio-Rad)”

With this second revised version, we hope that this manuscript is now ready for a publication in PLOS ONE *journal* and would be of interest for the scientific community.

Sincerely yours

Micheline Fromont-Racine
